# 

**Published:** 1919-06

**Authors:** 


### ANNOUNCEMENTS

### Officers, Michigan State Dental Society

The Michigan State Society, at the morning session, Wednesday, elected the following officers:

President, George F. Burke, of Detroit; president-elect C. G. Bates, of Durand; vice-president, Russell Bunting, of Ann Arbor; secretary, C. S. Larned, of Battle Creek; treasurer, E. J. Chamberlain, of Grand Rapids.

### "Build Now" Movement

The United States Department of Labor is urging every one who contemplates building, to begin as soon as possible in order that unemployed returning soldiers may find needed work.

*Production Depends on Construction.* Cutting down production, producing less food, less clothing, less fuel, never has and never will reduce prices. Increase production is the answer to the question of how we can reduce living costs.

We must have production if we are to have lower living costs. In modern industry production is stimulated by construction. Building is a basic industry. If you build a home you make business for more than a hundred correlated and contributing industries. Each building erected sends its wave of demand through the industrial organization, from the ditch-diggers to the bank president and back, and, by way of pay envelopes, to the ditch-digger again.

An immediate resumption of building activities will do more than any one thing to stimulate general business and accelerate the transition of industry from war demobilization to the peace normal.

Construction stimulates production—it creates the demand—and production must increase until it reaches the volume production necessary to decrease unit costs before prices are lower.

Assist business and serve your own and the nation's interests—*build now.*

---

### Program for Section on Stomatology of the American Medical Association, Atlantic City June 11-13, 1919

Wednesday, June 11—9 a. m. Meeting place—Royal Palace, Hall B. (1) Chairman's Address, Eugene S. Talbot, Chicago; (2) Macrocheilia, with Report of Two Cases (lantern demonstration), F. B. Moorehead, Chicago; (3) Teaching the Principles of Maxillofacial Surgery in a Civilian School, Charles R. Turner, Philadelphia; (4) Teaching the Principles of Maxillofacial Surgery in a Military School, G. V. I. Brown, Milwaukee; (5) Experience of a Dental Surgeon in an Evacuation Hospital, Rae P. McGee, Denver; (6) Experience of a Dental Surgeon in a Base Hospital in the Advanced Area, Ivan Smith, Mishawaka, Ind.

Thursday, June 12—9 a. m. Meeting place—Royal Palace, Hall B. Election of officers. (7) Experience of a Dental Surgeon in a Base Hospital in a Base Section, Stewart Ruggles, Portsmouth, Ohio; (8) Experience of an Area Consultant in the Zone of the Advance, George W. Schaeffer, Columbus, Ohio; (9) Experience of an Area Consultant in a Base Section, Justin M. Waugh, Hood River, Ore.; (10) Experience of an Area Consultant in the Intermediate Section, Herbert A. Potts, Chicago; (11) Observations on the Work at Queens Hospital in England, George M. Dorrance, Philadelphia; (12) Reconstruction Work in War Injuries of the Jaws (lantern demonstration), Robert H. Ivy, Milwaukee, and Joseph D. Eby, Washington, D. C.

Friday, June 13—9 a. m. Meeting place—Royal Palace, Hall B. (13) Bone Grafting in Jaw Cases, Frank J.

Tainter, St. Charles, Mo.; (14) Infected Fractures of the Mandible, Daniel H. Macauly, Jr., and Ernest P. Dameron, Cape May, N. J.; (15) Osteoperiosteal Bone Grafts of the Mandible as Performed by the French; A Report of Two Recent Cases, Henry S. Dunning, New York; (16) Prosthetic Appliances in Relation to the Surgical Treatment of Wounds of the Face and Jaws, V. H. Kazanjian, Boston; (17) Jaw Service at the American Red Cross Hospital No. 1, Paris, William Coughlin, St. Louis. General discussion of Oral Surgery Service in the War will be opened by Vilray P. Blair, St. Louis.

---

### Preparedness League Notes

*"Where There Is No Vision, the People Die."* These words of the prophet seem to have been written especially for the dental profession. If we have no vision we must die. The great things that are immediately before us are merely awaiting our vision and effort to make them practical realities.

The call of progress and humanity is ringing from end to end of the civilized world as never before. As a profession we must appreciate this and through it derive the benefits from the war which are within our grasp. Our erperience in the last few months in endeavoring to put forward different plans with this object in view has been quite discouraging. We seem to have dismissed the whole matter from our minds and are confining our energies to our own selfish interests. For the sake of our profession's future, let us carry the banner of *service* to the highest peak of mastery, that it may be seen and known throughout the world.

*Our Chance to Befriend Our Brother Dentists.* Our brother dentists of devastated France and Belgium need immediate help. The Germans drove them from their offices, appropriated their equipment, separated them from their families, and in many cases completely destroyed their towns.

A majority took up arms and fought in the trenches, many of whom were killed, while many more were partially or wholly incapacitated.

There are hundreds of cases where the wives and children of our brother dentists have suffered unspeakable tortures and are now without means of sustenance and support.

*Let the Dental Profession Take Care of Its Own.* These dentists are our brothers! Their trials, ambitions, hopes and lives have been the same as ours. Through the ravages of the diabolic Hun they are today without means to help themselves. Will we not stretch forth a helping hand? Will we not give them a brotherly and fraternal handclap and put into their exhausted spirit renewed hope, renewed ambition?

Place yourself in their position and think what assistance would mean to you, especially when it comes from a source with which your lifework has been identified.

Let the contributions of American dentists be their means of rehabilitation! Is it not a glorious thought? Let us muster our forces and carry forward to success this splendid object.

Following is an excerpt from a letter from Dr. Georges Villain of Paris: "The dentists of the North of France and Belgium have suffered terribly during the war. The Germans have removed and taken to Germany or destroyed all their material. Other dentists, half of the French practitioners, have left their offices since August, 1914 and will have to build over their practice. Some have been wounded to an extent that will oblige the use of special outfits to enable them to carry on their professional activities. Others are mutilated and will not be able to practice any more.

"Our professional brothers have given their lives, their limbs or their wealth, which amounted sometimes only to the value of their material, for the cause of liberty and freedom of the world, which is the cause of every one of us; what they have given we benefited, that is why we can not do too much. France has so much to do and every one over there suffered so much that we can not expect much better results than the one obtained.''

Through the marvelous skill of Dr. Villain and associates, fully 250,000 men who were injured by the war have been dentally restored and duly returned to the fighting lines.

In 1915 the Society l'Aide Confraternelle was organized by French and Belgian dentists for the following purposes:

''(1) To facilitate the return to normal life of all colleagues severely injured by the war, by giving to them or advancing as an honorable debt the necessary means to re-establish themselves; and

''(2) In the invaded districts to help in the reconstruction of destroyed homes and in newly-equipped dental offices devastated by the enemy, so as to enable dentists to resume their practice.''

A little more than $20,000 was raised, which amount was wholly inadequate to meet the demands. Our brother dentists of France have exhausted their resources and can not do more without aid from us.

All funds raised by the Preparedness League of American Dentists will be sent to the Treasurer of l'Aide Confraternelle, M. Fontanel, Paris, France, and judiciously disbursed according to the most worthy and pressing needs. A committee of American dentists in France will assist in directing the work.

A recent letter from Dr. E. C. Kirk states: ''I have just returned from Europe, and I can assure you that there is abundant need for every item of help we can render in the rehabilitation of our colleagues who have lost everything in the war-devastated areas.''

Lieutenant-Colonel Laflamme (successor to Colonel Logan in the Surgeon General's office) states: Your communication having reference to the furnishing of equipment for the French and Belgian dentists received. Wish to inform you I can safely and frankly state this office will, as in the past, be ready to support any movement that has for its end such lofty and generous ideas as mentioned in your letter.''

Now is the time to show a true spirit of helpfulness were most needed. A strict accounting will be made to all contributors and every protection afforded the handling of funds.

Make all checks payable to Dr. L. M. Waugh, Treasurer, Preparedness League of American Dentists' Fund for French and Belgian Dentists, 576 Fifth Avenue, New York, N. Y.

The following is an extract from a letter from Captain Blake A. Sears, Replacement Depot, Second Army, American Expeditionary Forces, France:

''As for the Belgian dentists, from all that I can learn, they are anxious for some one to finance them and let them alone. At the present time many of these dentists are sending their patients to the American camps for the necessary dentistry, and telling their patients that they can not do the work, as they can not purchase materials.

''The French dentists in this sector are not doing any dentistry. They just say that they have all they can do to get food at present without thinking of practicing dentistry, as the people are without the necessities and can not have work done. Simply, the teeth have to ache and then they are pulled. It is a case of where life does not depend upon one tooth and they are willing to sacrifice many as long as they can get food. Clothes are not up to date. Many are wearing discarded clothing from the armies, and they are not particular whether the trousers are from the French and the coat from the German, as long as they are warm. I have seen some of the women wearing hobnailed shoes from the U. S. Army, coats from the Italian, hats from the German, coats from the British, but the greater the variety of clothing the better they appear to like it.''—J. W. Beach, *President.*

